# VAliBS: a visual aligner for bisulfite sequences

**DOI:** 10.1186/s12859-017-1827-1

**Published:** 2017-10-16

**Authors:** Min Li, Ping Huang, Xiaodong Yan, Jianxin Wang, Yi Pan, Fang-Xiang Wu

**Affiliations:** 1 School of Information Science and Engineering, Central South University, Changsha, 410083 China; 2 Department of Computer Science, Georgia State University, Atlanta, GA 30302-4110 USA; 3 Division of Biomedical Engineering and Department of Mechanical Engineering, University of Saskatchewan, Saskatoon, SK S7N 5A9 Canada

**Keywords:** DNA methylation, Bisulfite mapping, Visual alignment

## Abstract

**Background:**

Methylation is a common modification of DNA. It has been a very important and hot topic to study the correlation between methylation and diseases in medical science. Because of the special process with bisulfite treatment, traditional mapping tools do not work well with such methylation experimental reads. Traditional aligners are not designed for mapping bisulfite-treated reads, where the un-methylated ‘C’s are converted to ‘T’s.

**Results:**

In this paper, we develop a reliable and visual tool, named VAliBS, for mapping bisulfate sequences to a genome reference. VAliBS works well even on large scale data or high noise data. By comparing with other state-of-the-art tools (BisMark, BSMAP, BS-Seeker2), VAliBS can improve the accuracy of bisulfite mapping. Moreover, VAliBS is a visual tool which makes its operations more easily and the alignment results are shown with colored marks which makes it easier to be read. VAliBS provides fast and accurate mapping of bisulfite-converted reads, and a friendly window system to visualize the detail of mapping of each read.

**Conclusions:**

VAliBS works well on both simulated data and real data. It can be useful in DNA methylation research. VALiBS implements an X-Window user interface where the methylation positions are visual and the operations are friendly.

## Background

Cytosine in CG dinucleotide (C in the 5′ end, G in the 3′ end) can be converted into 5-methyl cytosine under the enzyme by adding a methyl, which is called cytosine methylation of DNA. Cytosine methylation widely influences the expression of genes. Recent researches have shown that methylation is associated with many diseases, such as cancer, and methylation is heritable, which can be passed on to children from their parents [1]. One popular method in cytosine methylation research is bisulfite treatment.

**Fig. 1:**
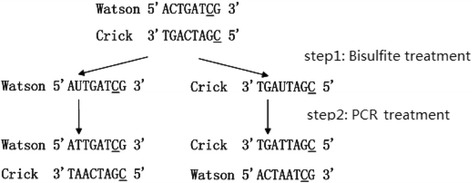
Bisulfite treatment (un-methylated cytosines converted to uracils (U)) and PCR treatment (U converted into thymine (T), four distinct strands: bisulfite Watson, bisulfite Crick, reverse com-plement of bisulfite Watson, and reverse complement of bisulfite Crick)

As shown in Fig. 1, in order to obtain methylation information, the DNA was dissolved into two single strands, where the underlined letter C marked the methylated cytosine. After bisulfite treated, non-methylated cytosine (C) will convert into uracil (U). Then PCR makes U converted into thymine (T), at the same time a double strand is synthesized based on each single strand (as shown in step 2 of Fig. 1). Different from normal mapping, the bisulfite mapping allows T to match C and A to match G in the reference.

By comparing un-bisulfite-treated to bisulfite-treated sequences, we can identify where cytosine is methylated. It has been shown by Deng et al. [2] that targeted bisulfite sequencing reveals changes in DNA methylation associated with nuclear reprogramming. Bisulfite conversion of genomic DNA combined with next-generation sequencing has been widely used to measure the methylation state of a whole genome and the study of complex diseases, such as cancer. A survey for analyzing the cancer methylome through targeted bisulfite sequencing is reported in reference [3]. Now the genome-wide bisulfite sequencing can also be used in single-cell [4], which provides a robust platform for molecular diagnotics [5]. Gu et al. optimized bisulfite sequencing and analyzed clinical samples with genome-scale DNA methylation mapping at single-nucleotide resolution [6]. Thus, it is of great interest to find the correct positions of bisulfite reads.

Recent years, great progresses have been made in the mapping tools for un-bisulfite-treated sequences [7]. Several tools have been developed including Bowtie [8], Bowtie2 [9], BWA [10], RAUR [11], etc., which have been used widely in the genome assembly [12, 13], contig error correction [14] and structural variation detection [15]. The existing mapping tools for bisulfite-treated sequences can be categorized into two groups: wild-card aligners and three-letter aligners [16, 17]. The common character of wild-card aligners is to replace cytosines in the sequenced reads with wild-card Y nucleotides to allow bisulfite mismatches. BSMAP [18], RMAPBS [19], GSNAP [20], and Segemehl [21] all employed this strategy. BSMAP was developed by Xi et al. based on a modified version of a general mapping tool SOAP [22]. BSMAP [18] adopted hashing and fast lookup methods to the octamer seeds converted from the reference genome and used a bit-mapping strategy to highlight mismatches from methylation and sequencing errors. RMAPBS [19] was developed by Smith et al. based on the RMAP program for mapping single-end bisulphite reads. GSNAP [20] was developed by Wu et al., which can be used for both single- and paired-end reads mapping and can detect short- and long-distance splicing, including interchromosomal splicing.

On the other hand, three-letter aligners, such as bsmapper (https://sourceforge.net/projects/bsmapper/), BS-Seeker [23], Bismark [24], BRAT [25], BRAT-BW [26] and MethylCoder [27], convert C to T in both sequenced reads and genome reference prior to performing the reads mapping by using modified conventional aligners. Bismark [24] was developed by Krueger et al. based on the mapping tool Bowtie2 [9], which was not only for bisulfite sequence mapping but also for methylation call. Three-letter strategy makes it easier to reuse non-bisulfite aligner as an internal module, with these non-bisulfite aligners improved, it is convenient to replace the internal module. BRAT-BW [26] developed by Harris et al. is a fast, accurate and memory-efficient mapping tool which maps the bisulfite-treated short reads by using FM-index (Burrows-Wheeler transform). MethylCoder [27] developed by Pedersen et al. is a flexible software tool for mapping bisulfite-treated short reads, which supports both paired- and single-end reads in color space or nucleotide formats. MethylCoder provides the option to user with two existing short-read aligners: Bowtie [8] and GSNAP [20].

Most of the three-letter aligners are fast, accurate, memory-efficient, and flexible. They are based on the modified conventional aligners and have been widely used. So, we believe that new tools for bisulfite-treated sequences with higher recall and precision could be implemented with the development of general mapping tools. In this paper, we developed a new tool VAliBS based on the three-letter strategy for mapping bisulfite-treated short reads by integrating two latest excellent mapping tools of Bowtie2 [9] and BWA [10]. Moreover, VAliBS is a visual tool, in which the alignment results are shown with colored marks which make it easier to be read.

## Methods

**Fig. 2:**
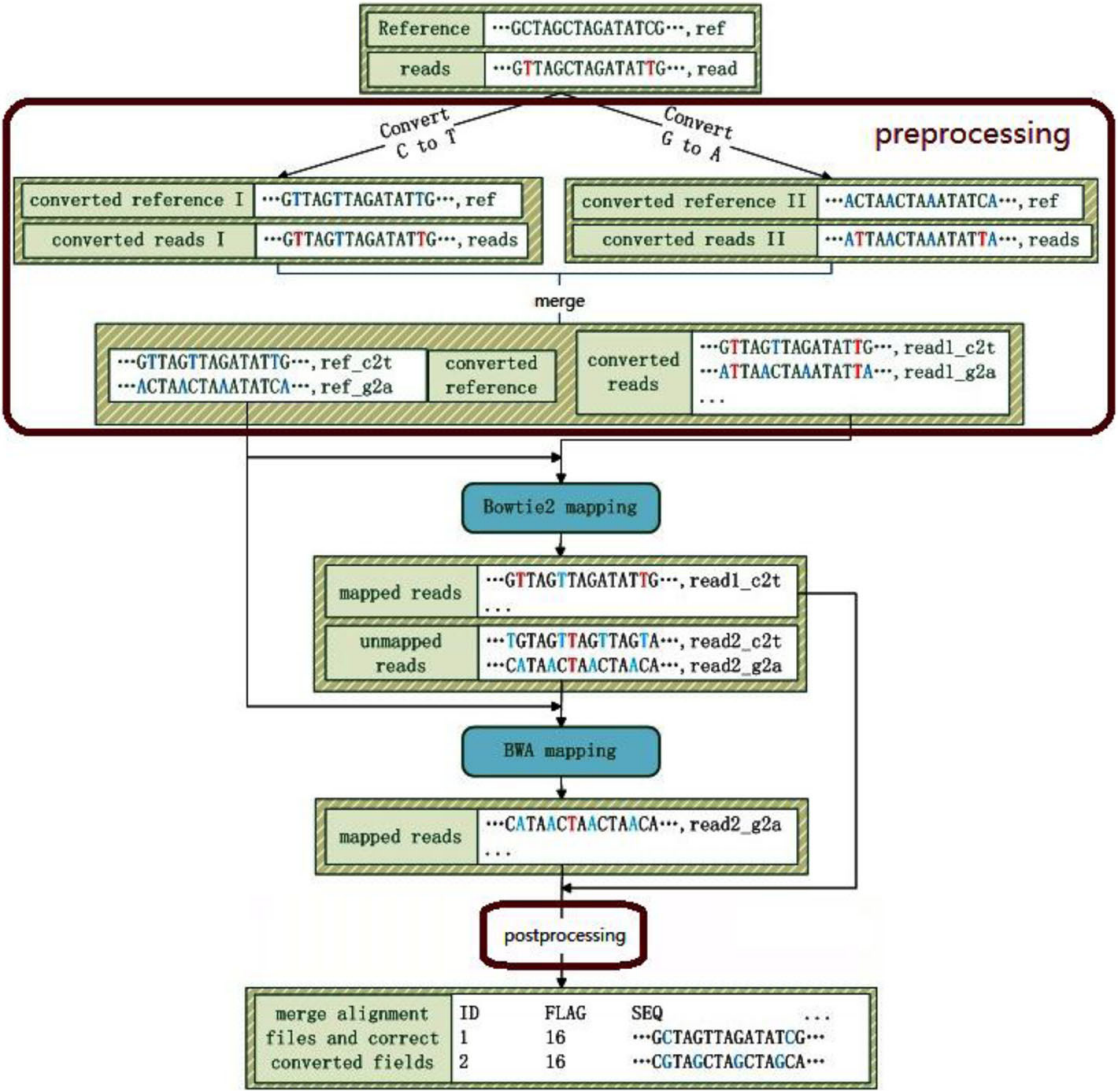
Schematic diagrams of VAliBS (pre-processing, mapping, and post-processing)

VAliBS has three stages: pre-processing, mapping, and post-processing. The schematic diagrams of VAliBS is shown in Fig. 2. In the following subsections we will introduce the three stages in detail.

### Pre-processing

According to Fig. 1,we know that the sequenced reads are bisulfite treated, and the reference is un-bisulfite treated. In the case that maps the reads to references directly without any processing, converted base positions will be regarded as mismatches and result in large scale match failure. To avoid these cases, we employee the widely used three-letter strategy. Three-letter strategy will mask the difference between bisulfite converted and un-bisulfite converted bases. Specificly, it masks the difference between C and T artificially, which in the other strand is G and A. As a result, for every reference, we make two copies for it, one converting all C to T, the other one converting all G to A; for every read, we conduct the same process. Now we get double references and reads and could observe that the conversion takes some pseudo mapping. For example, because C and T have no difference in the mapping process, read AGACCCATG is mapped into AGATTTATG on reference by mistakes. However, according to the methylation process, there only exists C-to-T conversion, and does not exist T-to-C conversion. These issues can be addressed in the post-processing stage. In the pre-processing, a conversion operation was implemented both for the genome reference and for the sequencing reads. Since C turns into T in the original strands of bisulfite-treated reads and G turns into A on the new reverse complementary strands, we hence use two types of base conversions: one is converting C to T, and the other is converting G to A.

### Mapping

Subsequently, the converted genome reference and the bisulfite-treated reads can be implemented on any one of the traditional mapping tools, such as SOAP [22], Bowtie2 [9], and BWA [10]. In this paper, we use two excellent mapping tools of BWA and Bowtie2, and integrate them into our tool VAliBS, as shown in Fig. 2. This integration is not mandatory, users can only choose one tool by optioning parameters. To integrate them effectively, we analyze their mapping results by using simulated datasets. The raw reads are simulated by ART [28] from hg19 chr22, and C or G in each read was converted randomly according to the known human DNA methylation level [29]. At last, two datasets of Illumina simulated bisulfite reads with 75 bp and 100 bp were obtained. The analysis results are shown in Table 1.

**Table 1:** Overlap of mapping rate between Bowtie2 and BWA on Illumina reads

Mapping Tools	Illumina 75 bp	Illumina 100 bp
Bowtie2	9380	8676
BWA	9205	8836
Overlap	8799	7968

From the analysis results we can see that Bowtie2 works very well on low-noise data, but has a lower recall for high-noise data, and BWA employs a heuristic method and always returns a high recall both on the low and high-noise data. Thus, we first use Bowtie2 to get a very reliable mapping set and then use BWA to the un-mapping reads. On the other hand, tools like Bowtie2 and BWA execute bi-directional mapping by default. It means that they try to map the reverse and complementary strands of reads into the reference. After the three-letter conversion, we expect to have the direction of mapping, we just want to see read_c2t (reads only contain A,T,G) mapping into reference_c2t (reference also only contains A,T,G) forward, not except the read_c2t (reads contain A,C,G) also mapping into refernceence_c2t after reverse and complementary conversion, i.e., read_c2t will map into reference_c2t only if read_c2t and reference_c2t are in the same strand. Therefore, we should forbid the optional of automatic bi-directional mapping. Moreover, to ensure no possible mapped reads are missed, we try to keep more mappings even those of false mappings. Actually, these false mappings will be filtered in the post-processing.

### Post-processing

**Fig. 3:**
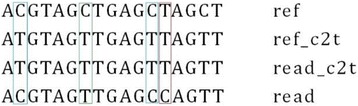
Example of error match by converting C to T. The positions marked with blue means methylated, because C in reads remain unchanged after bisulfite treatment. Positions marked with green means unmethylated. They converted to T after bisulfite treatment. Positions marked with red means false matching introduced after three-letter conversion. It should be a mismatch, because T can’t be converted to C

In the post-processing, we have implemented a procedure for filtering out most of mapping mistakes from the base conversion. As shown in Fig. 3, the positions marked with blue means methylated, because C in reads remains unchanged after bisulfite treatment. Positions marked with green means unmethylated. They converted to T after bisulfite treatment. Positions marked with red means false matching introduced after three-letter conversion. It should be a mismatch, because T can’t be converted to C.

In the post-processing, we also consider the mismatches with SNP tolerance by inputting SNP files to avoid filtering correct results. In addition, we need to merge the mapping results of Bowtie2 and BWA. Due to the introduction of conversion operation in VAliBS, it may generate multiple mapping results for the same original unconverted read. The repeated results will be removed.

### Visualization

**Fig. 4:**
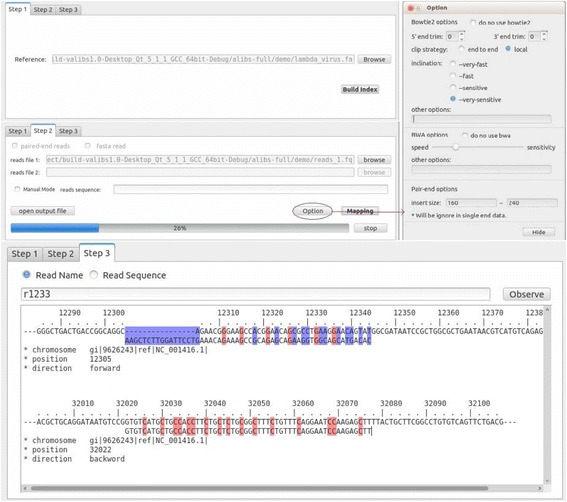
An example of visualization of VALiBS (operations and mapping results)

VAliBS is a visual tool for bisulfite sequence mapping. Distinguished from the previously command line tools, all of the operations of VAliBS can be implemented by using mouse. More importantly, a user can see how well a read is mapped to the genome reference. The mapping results are marked with colors, the insertions, deletions and mismatches are marked with blue while the methylation bases were marked with red. An example was shown in Fig. 4. If one read has multiple mapping results, it can also be displayed in the same window.

## Results and Discussion

### Experimental data

In order to validate the effectiveness of VAliBS, we compare it with other popular bisulfite mapping tools: Bismark [24], BS-Seeker2 [30], and BSMAP [18]. VAliBS, Bismark, and BS-Seeker2 are all the three-letter-based approaches. Bismark [24] is an efficient bisulfite mapping tool based on the modification of Bowtie2. BS-Seeker2 [30] is an updated version of BS-Seeker, which further improves the mappability by using local alignment. BSMAP [18], on the contrast, is a method based on the wild-card approach. We compared them on both the simulation data and the real data.

The simulation data and real data are used as the same as in BSSeeker2 [30]. Since our tool VAliBS for RRBS data did not have special treatment, we did not test RRBS data. Only WGBS data was used in our experiments. Two kinds of simulated sequences (error-free and error-containing) were used. For each kind of simulated sequences, both single-end and paired-end data were generated. The simulated error-containing sequences were converted with 1% failure, to which the sequencing errors by cycles were also added [30]. The error-free simulated sequences were converted faithfully with no sequencing error. The single end of real data was from the published data sets, SRR299053 (mouse) and the paired-end of real data was from SRR306438 (human) [31].

### Performance on simulation data

The comparison results of VAliBS, Bismark, BS-Seeker2, and BSMAP on the simulation data were shown in Table 2. Here we evaluated the performance of these four bisulfite mapping tools by using mappability and correct mappability.

**Table 2:** Comparison of VAliBS, Bismark, BS-Seeker2, and BSMAP on simulation data

single end	VAliBS	BS-Seeker2		Bismark		BSMAP
	bowtie2	Bowtie2	Bowtie	Bowtie2	Bowtie	
Simulation: error-free						
map	92.80%	91.50%	91.65%	87.78%	91.65%	91.81%
c-map	92.09%	91.50%	91.65%	87.78%	91.65%	91.81%
Simulation: error-containing						
map	92.67%	90.51%	91.69%	86.90%	91.64%	91.90%
c-map	91.23%	90.26%	91.59%	86.79%	91.46%	91.82%
paired end	VAliBS	BS-Seeker2		Bismark		BSMAP
	Bowtie2	Bowtie2	Bowtie	Bowtie2	Bowtie	
Simulation: error-free						
map	92.79%	78.02%	78.29%	72.51%	78.08%	78.63%
c-map	92.08%	78.02%	78.29%	72.51%	78.08%	78.49%
Simulation: error-containing						
map	94.24%	78.42%	78.72%	71.36%	78.17%	79.10%
c-map	92.64%	77.07%	77.95%	71.08%	77.25%	78.16%

The mappability (abbreviated as map in Table 2) is defined as the percentage of reads that are uniquely mapped over all reads. The correct mappability (abbreviated as c-map in Table 2) is defined as the percentage of corrected unique mapping.

VAliBS integrated Bowtie2 and BWA, which has greater flexibility and obtains different results with different parameters. As both Bismark and BS-Seeker2 used Bowtie2, we listed the results of VAliBS only by using Bowtie2. For comparison, the recommended parameters of Bowtie2 were used to evaluate the mappability and correct mappability of VAliBS, Bismark, and BS-Seeker2.

From Table 2 we can see that VAliBS, Bismark, BS-Seeker2, and BSMAP all work well on the single-end data for both error-free and error-containing data. Compared to the application on the simulated error-free data, the mappability and correct mappability of all the four bisulfite mapping tools slightly descend when being applied on the simulated data with noise. When being applied on the paired data, the mappability and correct mappability of VAliBS are much higher than those of Bismark, BS-Seeker2, and BSMAP.

### Performance on real data

VAliBS, Bismark, BS-Seeker2, and BSMAP were all tested on the real data. The comparison results were shown in Table 3. As for the real data, we do not know whether the unique mapping is correct or not. Only the mappability is calculated and compared. From Table 3 we can see that the mappability of VAliBS is consistently higher than that of Bismark, BS-Seeker2, and BSMAP both for single-end data (SRR299053/mouse) and paired-end data (SRR306438/human).

**Table 3:** Comparison of VAliBS, Bismark, BS-Seeker2, and BSMAP on single-end data (SRR299053/mouse) and paired-end data (SRR306438/human)

mappability	VAliBS	BS-Seeker2		Bismark		BSMAP
	Bowtie2	Bowtie2	Bowtie	Bowtie2	Bowtie	
single end	82.88%	72.94%	71.89%	70.31%	73.15%	72.84%
paired end	56.64%	48.78%	47.29%	44.24%	46.89%	45.64%

### Feature comparisons

VALiBS supports many features, which can meet most of environments, as shown in Table 4. VALiBS supports Illumina and 454 platform’s reads,quality or no-quality reads format (FASTA/Q), indel and gap, allowing mapping both single end and paired-end reads. Its output format is the widely used format SAM, to facilitate subsequent steps. The most important feature of VALiBS is visualization, which can be operated intuitionally. Not only the process operations, but also the mapping results can be visualized. A comprehensive comparison of VAliBS, Bismark, BS-Seeker, BS-Seeker2, and BSMAP is also shown in Table 4.

**Table 4:** Features supported by Bismark, BS-Seeker, BS-Seeker2, BSMAP, and VAliBS

Aligners	Bismark	BS-Seeker	BS-Seeker2	BSMAP	VAliBS
O.S.	Linux,Mac	Linux,Mac	Linux, Unix, Mac	Linux, Unix, Mac	Linux
Seq.Plat.	I	I	I	I	I, 4
Input	FASTA/Q	FASTA/Q	FASTA/Q, qseq	FASTA/Q SAM/BAM	FASTA/Q
Output	SAM	SAM	SAM/BAM	SAM BAM Native	SAM
Min. RL	16		10	20	22
Max. RL	10 K		200	144	
#Mis	Score	3	Score	15	Score
Indels	Score	0	Score	1	Score
Gaps	N	N	Y	N	Y
Align. Reported	U	U	B,U,S	B,R,U	A,B
Alignment			G, L	G	G, L
Parallel	SM	SM	SM	SM	SM
QA	Y	Y	N	N	Y
PE	Y	Y	Y	Y	Y
Vis	N	N	N	N	Y

Abbreviations in Table 4 are as following: 1) Sequencing Platform: I-Illumina; So-ABI Solid; 4-Roche 454; Sa-ABI Sanger; 2) Read Length: K denotes kilobases (1000 bases); M denotes meg-abases (1000 K bases); and * denotes a (unknown) large number; 3) Alignments reported: A-all, B-best; R-random; U-unique alignments only (no multimaps); S-user defined number of matches; 4) Alignment: G-(semi-)global (a.k.a. end-to-end); L-Local; 5) Parallelism: SM-shared-memory; DM-distributed memory; Cloud - Cloud computing; 6) Vis: visualization; 7) Y-Yes; N-No

## Conclusions

DNA methylation is very important to the research of diseases. In this paper, we have designed and implemented a visual tool VAliBS for bisulfite sequence alignment based on base conversions. VAliBS is fast, memory-efficient and reliable, which can be useful in DNA methylation research. More importantly, VAliBS is a visual tool where the alignment results and the methylation positions are visual while the operations are friendly. In addition, pre-processing and post-processing are decoupled with Bowtie2 and BWA, to make them easily updating modularity. As MapReduce frame has been used widely in bioinformatics [32], the efficiency performance of VAliBS can even be improved by parallel processing in the future.
